# Integrative taxonomy of the ornamental ‘peppermint’ shrimp public market and population genetics of *Lysmata boggessi*, the most heavily traded species worldwide

**DOI:** 10.7717/peerj.3786

**Published:** 2017-09-18

**Authors:** J. Antonio Baeza, Donald C. Behringer

**Affiliations:** 1 Department of Biological Sciences, Clemson University, Clemson, SC, United States of America; 2 Smithsonian Marine Station at Fort Pierce, Fort Pierce, FL, United States of America; 3 Departamento de Biologia Marina, Universidad Catolica del Norte, Coquimbo, IV Region, Chile; 4 Program in Fisheries and Aquatic Sciences, University of Florida, Gainesville, FL, United States of America; 5 Emerging Pathogens Institute, University of Florida, Gainesville, FL, United States of America

**Keywords:** Peppermint shrimp, Dna profiling, Barcoding, Forensic genetics, *Lysmata*

## Abstract

The ornamental trade is a worldwide industry worth >15 billion USD with a problem of rampant product misidentification. Minimizing misidentification is critical in the face of overexploitation of species in the trade. We surveyed the peppermint shrimp ornamental marketplace in the southeastern USA, the most intense market for peppermint shrimps worldwide, to characterize the composition of species in the trade, reveal the extent of misidentification, and describe the population genetics of the true target species. Shrimps were bought from aquarium shops in FL, GA, SC, and NC. We demonstrated, contrary to popular belief (information from dealers), that the most heavily traded species in the market was *Lysmata boggessi*, an endemic species to the eastern Gulf of Mexico, and not *Lysmata wurdemanni*. Importantly, only when color pattern or genetic markers in conjunction with morphological traits were employed, was it was possible to unequivocally identify *L. boggessi* as the only species in the trade. The intensity of the market for peppermint shrimps in the USA has led to *L. boggessi* being the most traded species worldwide. Misidentification in the shrimp aquarium trade is accidental and involuntary, and is explained by remarkable similarity among congeneric species. Using sequences of the 16S-mt-DNA marker, we found no indication of population genetic structure in the endemic *L. boggessi* across  550 km of linear coast. Therefore, this species can be considered genetically homogeneous and a single fished stock. Still, we argue in favor of additional studies using more powerful markers (e.g., SNPs) capable of revealing genetic structure at a finer spatial-scale. Our results will help advance management and conservation policies in this lucrative yet understudied fishery. Future studies of other ornamental fisheries will benefit from using an integrative taxonomic approach, as we demonstrate here.

## Introduction

The aquarium trade is a large worldwide industry worth >15 billion USD (Bartley, 2000; Penning et al., 2009) that supplies collectors with an assortment of >6300 species of algae, plants, invertebrates, and vertebrates (Chapman et al., 1997; Calado et al., 2003; Bruckner, 2005; Rhyne et al., 2009). The aquarium trade shares two characteristics with various other industries that rely on the exploitation of natural resources; sources of supply are diffuse and morphological identification of the species involved in the trade can be logistically challenging (because they are members of species complexes) or sometimes impossible (if the species is processed prior to sale). It is not surprising, therefore, that product misidentification is rampant in this industry, a major issue already detected in other industries that depend upon the extraction of ‘exotic’ natural resources such as marine turtles in the USA (Roman & Bowen, 2000), global whale meat market (Baker & Palumbi, 1994; Baker, Cipriano & Palumbi, 1996), sea horses in apothecary shops and curio stores (Sanders et al., 2008), black sturgeon caviar (DeSalle & Birstein, 1996), wild bush meat (Eaton et al., 2010), and several marine fisheries (‘Chilean sea bass’—Marko, Nance & Guynn, 2011; ‘European megrims’—Crego-Prieto et al., 2012; ‘hake’—Muñoz-Colmenero et al., 2015).

Thus, solving or minimizing the widespread misidentification problem is critical and particularly so in the aquarium trade. Correctly identifying the species in the trade might help in revealing illegal or unregulated exploitation of wildlife (e.g., Baker et al., 2002; Marko et al., 2004). The proper identification of species in the industry is a first step in revealing trade patterns that may require conservation measures, especially if the species involved in the trade are endangered (Sanders et al., 2008). Lastly, proper product identification permits the estimation of exploitation rates, reliable certification of products in the supply chain, and subsequent development of sustainable management guidelines (Baker, 2008). In the case of the aquarium trade, a proper description of the species involved in the market place is particularly relevant in the face of rising demand and increasing overexploitation (Green, 2003; Calado et al., 2003; Rhyne et al., 2009).

**Figure 1:**
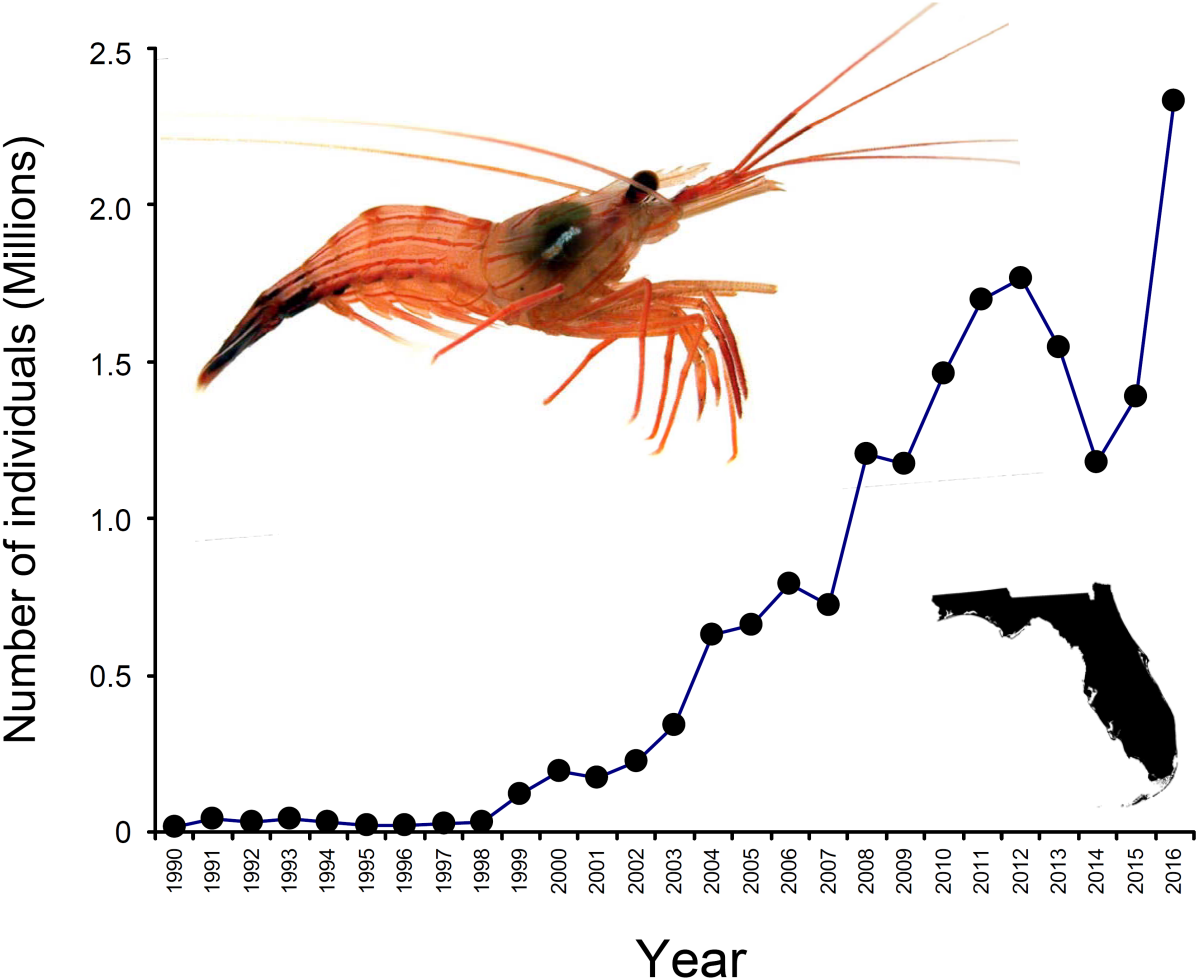
Number of peppermint shrimp captured per year for the period 1990–2016. Data from FWC, available at https://publictemp.myfwc.com/FWRI/PFDM/. These data rely on FWC Trip Ticket reports completed by fishermen and buyers at the time of sale. Notice that nearly 2.3 million individuals were harvested from Florida (inset on the bottom right) during 2016 alone. The shrimp on the top left is *Lysmata boggessi*.

In North America, the state of Florida is a hotspot for the harvesting of marine ornamentals (Rhyne et al., 2009). Landings of >9 million ornamental organisms, comprising >600 fish, invertebrates (e.g., shrimps, crabs, snails, anemones, corals), and plant species, are reported yearly from Florida alone (Rhyne et al., 2009). Among shrimps (infraorder Caridea), the genus *Lysmata* (peppermint shrimps) is the most heavily exploited and traded ((Calado, 2008; Rhyne et al., 2009; Baeza et al., 2014); Florida Fish and Wildlife Conservation Commission [FWCC]). *Lysmata* spp. are distributed worldwide, with much of the species diversity centered in the Indo-Pacific (Baeza, 2009; Anker & Cox, 2011), but exploitation of these shrimps is greatest in south Florida (Rhyne et al., 2009; Baeza et al., 2014; Prakash et al., 2017). Indeed, an estimated 2.3 million individuals harvested from Florida entered the aquarium trade during 2016 (FWCC data, available at https://publictemp.myfwc.com/FWRI/PFDM/, Fig. 1). Most peppermint shrimps are caught as bycatch either in stone crab *Menippe mercenaria* traps or in nets from roller-frame shrimp trawlers targeting bait or edible (penaeid) shrimps (Baeza et al., 2014).

Although highly sought after because of their aesthetic value and ability to control aquarium pests (Calado, 2008; Rhyne, Lin & Deal, 2004; Rhyne et al., 2009), little is known about the identity of the species involved in the trade. Historically, peppermint shrimps available at aquarium shops have been labeled and sold as *Lysmata wurdemanni* (Rhyne, Lin & Deal, 2004; Rhyne et al., 2009; Calado, 2008). However, *L. wurdemanni* is now recognized as a species complex comprising more than seven anatomically similar species (Rhyne, Lin & Deal, 2004; Rhyne & Lin, 2006; Baeza et al., 2009; Baeza, 2013). Thus, in addition to conservation benefits, proper identification of the species available in the market place has the potential to reveal cryptic new species (Von der Heyden et al., 2014). Ultimately, this effort should translate into improved management measures for this lucrative yet poorly regulated and understudied industry (Von der Heyden et al., 2014).

The aim of this study was two-fold. Our first goal was to survey the peppermint shrimp ornamental market place in the southeastern USA, the most intense and dynamic market for peppermint shrimps worldwide (Calado, 2008; Rhyne et al., 2009; Prakash et al., 2017). To characterize the composition of peppermint shrimp species involved in the trade, we used an integrative taxonomic approach, including molecular barcoding tools, morphological traits, and color attributes. The strategy above permitted us to determine which peppermint shrimp species in Florida are most heavily traded. Once we generated a snapshot of the market composition, our second goal was to explore the population genetics of the most important species in the trade. Inclusion of genetic research on this lucrative fishery will help advancing management policies and conservation measures, an objective proved to be challenging in other industries (Von der Heyden et al., 2014). Specifically, we tested for significant genetic differentiation in the most commonly traded peppermint shrimp species in the industry using sequences of the mitochondrial gene fragment 16S, a marker that is well suited for population genetic studies (Hellberg, 2009; Baeza & Fuentes, 2013a; Baeza & Fuentes, 2013b).

## Materials and Methods

### Sampling of peppermint shrimps from aquarium stores

Field collections were approved by FWCC (permit number: SAL-11-1319-SR). Dealer surveys were approved by Clemson University Institutional Review Board (Exempt Determination: IRB2017182; verbal consent).

We used an integrative taxonomic approach to identify the most traded species of peppermint shrimp in the aquarium trade and to reveal putative species misidentification in the ornamental industry. A total of 43 shrimps were bought from different aquarium stores (wholesalers = 3, retail stores = 8) located in Florida, Georgia, South Carolina, and North Carolina during the years 2014–2016. At each store, we asked three questions to the dealer: “Do you know the scientific name(s) of the peppermint shrimp(s) I am buying?”, “Do you know if the shrimp(s) I am buying are harvested from the wild or are produced at aquaculture facilities?”, and “If harvested, do you know which state or geographic locality they were harvested from?”. The answers to the three questions combined with the results of this study allowed us to address species misidentification in the peppermint shrimp market in the southeast USA.

Following purchase, each shrimp was transported alive to the laboratory at Clemson University, South Carolina, or to a field laboratory on Long Key, Florida, where the detailed morphology and color pattern of each shrimp was examined. Also, for a subsample of specimens (see below), we sequenced a fragment of mitochondrial DNA. Information on morphology, coloration pattern, and genetic characters were used in combination to draw inferences about the identity of the species traded at aquarium shops, while taking into account character variability.

### Coloration, color pattern and comparison with sympatric congeneric species

We examined the coloration and color pattern in the totality of the specimens bought at aquarium shops. The coloration and color pattern of all of the species present in the northwestern Atlantic, Gulf of Mexico, and Caribbean Sea are well known (Chace Jr, 1972; Chace Jr, 1997; Rhyne & Lin, 2006; Rhyne & Anker, 2007; Baeza & Anker, 2008; Baeza et al., 2009; Soledade et al., 2013). Each shrimp was examined while still alive, or a few minutes after fixation. We focused on the color pattern of the carapace (dorsal view), abdominal pleura (lateral view), uropods (dorsal view), and eggs (if present) carried underneath the abdomen. Previous studies have demonstrated that the color pattern in peppermint shrimps represents a reliable marker for species identification ((Rhyne & Lin, 2006; Baeza et al., 2009; Anker & Cox, 2011; Soledade et al., 2013), and references therein). For instance, *Lysmata rathbunae* has a distinct transverse V-shaped band on the third pleuron. This band is absent in closely related species i.e., *L. wurdemanni* and *L. pederseni* (Rhyne & Lin, 2006). Also, *Lysmata boggessi* bears a distinct inverted Y on the posterior of the carapace (dorsal view) that is absent in all closely related species (Rhyne & Lin, 2006; Baeza et al., 2009). Lastly, most species produce light to dark green eggs but *L*. *pederseni* is the only species in the region known to produce pink eggs (Rhyne & Lin, 2006). Considering that the species sold most commonly in aquarium stores is believed to be *L. wurdemanni* (see results), we focused particularly on determining whether or not the specimens we purchased belonged to this species.

### Morphological variation and comparison with congeneric sympatric species

After evaluating the color pattern, we recorded the following morphological characters: dorsal and ventral dentition of the rostrum, number of segments on the carpus and merus of the second pereopod, and the number of spiniform setae on the ventro-lateral margin of the merus and on the ventral margin of the propodus of pereopods 3-5. Using the diagnostic characters above, we compared the specimens bought from aquarium stores to all other species present in the greater Caribbean, Gulf of Mexico, and northwestern Atlantic. The specimens were identified using descriptions and re-descriptions by Chace Jr (1972), Rhyne & Lin (2006), Rhyne & Anker (2007), Baeza & Anker (2008), Baeza et al. (2009), Soledade et al. (2013), Rhyne et al. (2012), and the dichotomous keys of Chace Jr (1997) and Rhyne & Lin (2006). Here again, we particularly focused on determining whether or not the specimens were *L. wurdemanni*.

### Phylogenetic position and comparison with congeneric sympatric species

Detailed examination of the color pattern indicated that the aquarium store specimens belonged to a single species of peppermint shrimp (see results) different than *L. wurdemanni*. Therefore, we haphazardly choose a sub-sample of 25 specimens out of the 43 bought at aquarium stores to extract genomic DNA and determine their genetic identity. We conducted a molecular phylogenetic analysis using these 25 shrimps and 57 sequences of the following: three sequences belonging to specimens of *Lysmata pederseni*, two sequences belonging to specimens of *Lysmata udoi*, two sequences belonging to specimens of *Lysmata moorei*, two sequences belonging to specimens of *Lysmata udoi*, and one sequence each belonging to specimens of *Lysmata rafa*, *Lysmata bahia, Lysmata galapagensis, Lysmata ankeri*, *Lysmata jundalini*, *Lysmata* cf. *californica*, and *Lysmata hochi*. We also included 26 sequences belonging to *Lysmata wurdemanni*. Thirteen out of these 26 sequences were retrieved from Genbank while the remaining 13 sequences were generated during this study and represent the totality of haplotypes found in 46 individuals previously used for population genetic analysis in the Gulf of Mexico and eastern Florida (Rhyne et al., 2009; Baeza et al., 2014). Lastly, we included 16 sequences belonging to *Lysmata boggessi*. Three out of these 16 sequences were retrieved from Genbank while the remaining 13 sequences were generated during this study and represent haplotypes found among the 88 individuals collected to describe population genetics of *L. boggessi* (see section Population genetics of *Lysmata boggessi*). The species above represent all of the species from the genus *Lysmata* described for the Gulf of Mexico, northeastern Atlantic, and Caribbean Sea. The only species not included in the analysis was *Lysmata rathbunae* for which no sequences are available in Genbank. Lastly, two other sequences belonging to the scarlet cleaner shrimp *Lysmata grabhami* were included as out-groups during the first phylogenetic analysis. Accession numbers for sequences retrieved from Genbank are provided immediately after the species names.

In this analysis, we expected all aquarium store specimens (i) to cluster together and form a single monophyletic clade collectively with the newly generated haplotypes and (ii) to segregate from other sequences used in the first analysis if the aquarium stores specimens indeed belonged to *Lysmata boggessi* (see below).

### DNA extraction, amplification, and sequencing

We extracted total genomic DNA either from pereopods or from abdominal muscle tissue of the 25 shrimps bought at aquarium shops using the OMEGA BIO-TEK^®^ E.Z.N.A.^®^ Blood and Tissue DNA Kit following the manufacturer’s protocol. Next, PCR was used to amplify an approximately 557 base-pair (bp) region (excluding primers) of the 16S rRNA DNA, using the primers 16L2 (5′-TGC CTG TTT ATC AAA AAC AT-3′) and 1472 (5′-AGA TAG AAA CCA ACC TGG-3′) (Schubart, Neigel & Felder, 2000). Standard PCR 25-µl reactions (17.5 µl of GoTaq^®^ Green Master Mix [Promega^®^], 2.5 µl each of the two primers [10 mM], and 2.5 µl DNA template) were performed on a C1000 Touch™ Thermal Cycler (BIORAD^®^) under the following conditions: initial denaturation at 95 °C for 5 min followed by 40 cycles of 95 °C for 1 min, 56 °C for 1 min, and 72 °C for 1 min, followed by chain extension at 72 °C for 10 min. PCR products were purified with ExoSapIT (a mixture of exonuclease and shrimp alkali phosphatase, Amersham Pharmacia) and then sent for sequencing with the ABI Big Dye Terminator Mix (Applied Biosystems) to the Clemson University Genomics Institute (CUGI—Clemson University, Clemson. South Carolina), which is equipped with an ABI Prism 3730xl Genetic Analyzer (Applied Biosystems automated sequencer). All sequences were confirmed by sequencing both strands and a consensus sequence for the two strands was obtained using the software Sequencer 5.4.1 (Gene Codes Corp.).

### Sequence alignment and phylogenetic analyses

Sequence alignment was conducted using Multiple Sequence Comparison by Log-Expectation in MUSCLE (Edgar, 2004) as implemented in MEGA6 (Tamura et al., 2013). Next, the aligned sequences were analyzed with the software jModelTest 2 (Darriba et al., 2012), which compares different models of DNA substitution in a hierarchical hypothesis–testing framework to select a base substitution model that best fits the data. The optimal models found by jModelTest 2 (selected with the corrected Akaike Information Criterion [AIC_*c*_]) was a TVM+G evolutionary model. The calculated parameters were as follows: assumed nucleotide frequencies A= 0.3216, *C* = 0.1240, *G* = 0.2022, *T* = 0.3522; substitution rate matrix with A–C substitution = 0.2536, *A*–*G* = 5.6574, *A*–*T* = 1.0456, *C*–*G* = 0.8591, *C*–*T* = 5.6574, *G*–*T* = 1.0; and a gamma distribution (G) with shape parameter = 0.3120. This model was implemented in MrBayes (Huelsenbeck & Ronquist, 2001) for Bayesian Inference (BI) analysis and raxmlGUI version 1.5b1 (Silvestro & Michalak, 2012) for maximum likelihood (ML) analyses.

For BI, unique random starting trees were used in the Metropolis–coupled Markov Monte Carlo Chain (MCMC) (see Huelsenbeck & Ronquist, 2001; Ronquist et al., 2012). The analysis was performed for 6,000,000 generations. Visual analysis of log-likelihood scores against generation time indicated that the log-likelihood values reached a stable equilibrium before the 100,000th generation. Thus, a burn-in of 1,000 samples was conducted, every 100th tree was sampled from the MCMC analysis obtaining a total of 60,000 trees and a consensus tree with the 50% majority rule was calculated for the last 59,000 sampled trees. For the ML analysis, we use the option bootstrap + consensus tree in raxmlGUI. All the rest of the parameters used were those of the default option in raxmlGUI. The robustness of the ML tree topology was assessed by bootstrap reiterations of the observed data 1,000 times. Support for nodes in the BI tree topology was obtained by posterior probability.

### Population genetics of Lysmata boggessi

#### Shrimp collections and sampling rationale

Our results indicated that the species of peppermint shrimp traded at aquarium stores was not *L. wurdemanni* but *L*. *boggessi* (see results). Therefore, our next objective was to describe the population genetic structure of *L. boggessi* across its entire geographic range in the Gulf of Mexico.

A total of 88 *L. boggessi* were collected from five different localities along the Gulf coast of Florida: Sandy Key (25°02′11.3″N, 81°00′52.0″W), Cedar Key (29.1386°N, 83.0351°W), Hernando Beach (28.4694°N, 82.6593°W), Pavilion Key (25.6979°N, 81.3551°W), and Sawyer Key (24.7588°N, 81.5654°W), during 2015. We choose these localities to enable us to test for genetic dissimilarity among populations of this endemic species almost throughout its entire geographic range in the Gulf of Mexico. In all localities, shrimps (*N* = 15–21) were collected from stone crab traps deployed by fishermen in the shallow (<8 m) subtidal zone. After shrimps were retrieved from the traps, they were preserved in 95% ethanol. The species identity of each collected shrimp was confirmed using Chace Jr (1972; 1997) and the taxonomic key in Rhyne & Lin (2006), as well as the protocol explained above.

#### DNA extraction and sequence alignment

Tissue and total genomic DNA extraction, PCR amplification with specific primers, product cleanup, and sequencing were conducted as described above (see section *Sequence alignment and phylogenetic analyses*). All sequences obtained during this study were deposited in GenBank (accession numbers: MF632120–MF632134 and MF632135–MF632247).

#### Phylogeographic and population genetic analyses

The software POPART (available online at http://popart.otago.ac.nz) was used to estimate a haplotype network for the target gene fragment. This software implements, among others, the statistical parsimony procedure described in Templeton, Crandall & Sing (1992) and Crandall (1994) that provides a 95% plausible set of relationships among haplotypes (see also Clement, Posada & Crandall, 2000).

The software DnaSP version 5.10.1 (Librado & Rozas, 2009) was used to assess diversity at each sampling locale. The standard diversity indices herein calculated for each locality were number of haplotypes, haplotype diversity (Nei, 1987) and nucleotide diversity (per site, Tajima, 1983; Tajima, 1993; Nei, 1987). To test for genetic variance within and among populations, an analysis of molecular variance (AMOVA—Excoffier, Smouse & Quattro, 1992) was conducted in the software ARLEQUIN version 3.5.1.3 (Excoffier, Laval & Schneider, 2005) using uncorrected haplotype pairwise differences as a measure of divergence.

To explore the relationship between genetic and geographical distance in *L. boggessi*, a Mantel test and a reduced major axis (RMA) regression (after log–log transformation, see Slatkin, 1993) were conducted in the server IBDWS Version 3.23 (Jensen, Bohonak & Kelley, 2005). The significance of the Mantel test and RMA regression was determined with 20,000 permutations. Measurements of distances between pairs of localities in the Gulf of Mexico were taken using the ‘path ruler’ tool in Google Earth (http://earth.google.com/). For each pair, we measured the shortest distance avoiding islands and mainland land masses.

## Results

### The identity of peppermint shrimps traded in the aquarium industry

To the question “Do you know the scientific name(s) of the peppermint shrimp(s) I am buying?”, most dealers indicated that the species was *L. wurdemanni* (*N* = 7). Three dealers knew the genus to which the peppermint shrimps belong but did not know the species name. One dealer knew neither the genus nor the species name. To the question “Do you know if the shrimps I am buying are harvested from the wild or are produced at aquaculture facilities?”, most dealers answered that the specimens being sold were harvested (*N* = 10). One dealer did not know if the specimens being sold were harvested or produced at an aquaculture facility. Lastly, to the question “If harvested, do you know which state or geographic locality they were harvested from?”, most dealers answered that the specimens were harvested from Florida (*N* = 8). Two dealers knew that the specimens were harvested but did knot know from where.

### Morphological variation and comparison with sympatric species

**Figure 2:**
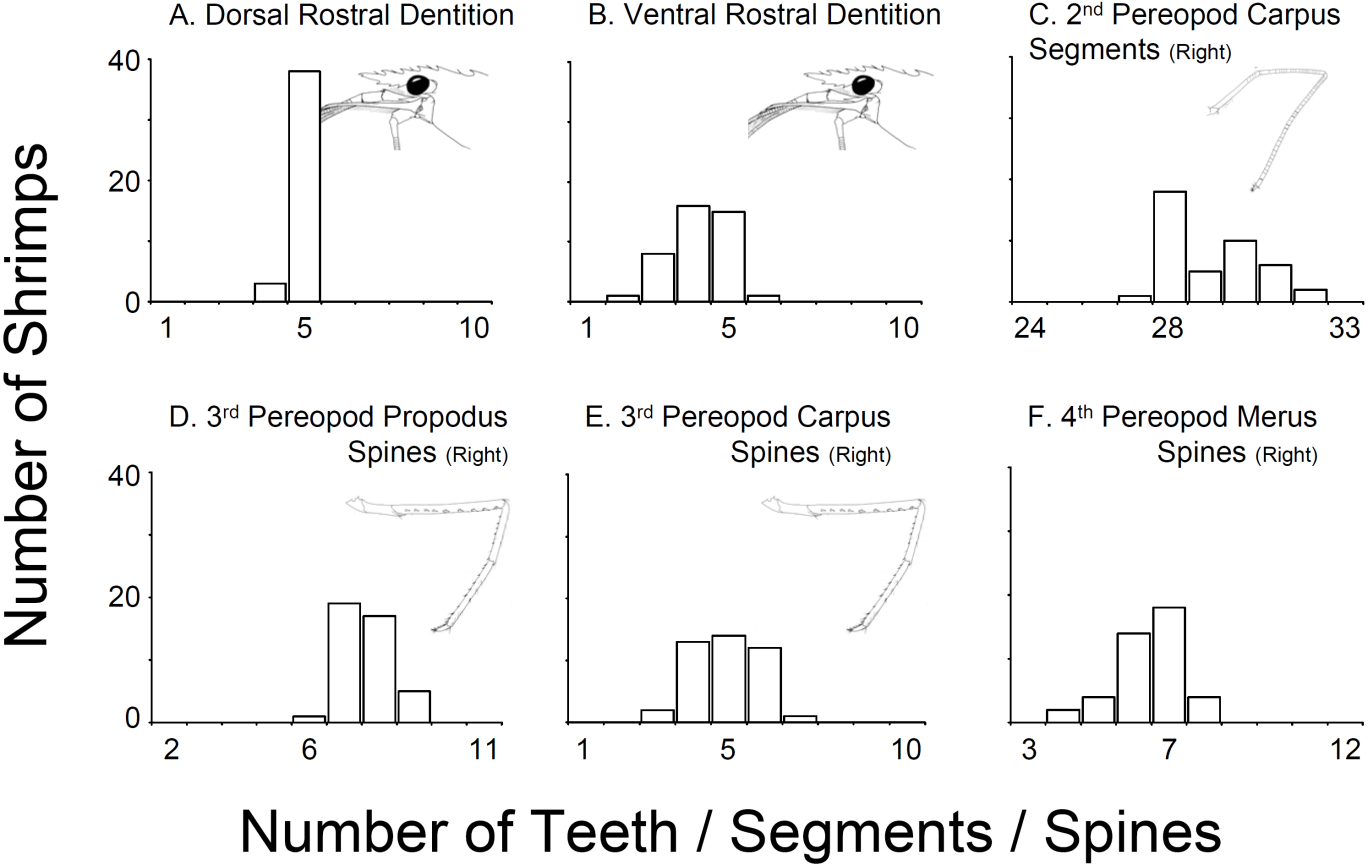
Variability in selected characters of systematic relevance in shrimps bought at aquarium stores in southeastern USA. (A) Dorsal Rostral Dentition, (B) Ventral Rostral Dentition, (C) 2nd Pereopod Carpus Segments (Right), (D) 3rd Pereopod Propodus Spines (Right), (E) 3rd Pereopod Carpus Spines (Right), (F) 4th Pereopod Merus Spines (Right).

In all 43 specimens bought from aquarium stores in Florida, Georgia, South Carolina, and North Carolina, the rostral dentition varied moderately (Table 1, Fig. 2). Most of the specimens analyzed possessed five dorsal and 5 ventral teeth (*n* = 15) or 5 dorsal and 4 ventral teeth (*n* = 13) or 5 dorsal and 3 ventral teeth (*n* = 8). A few specimens exhibited 5 dorsal and 6 ventral teeth (*n* = 1), or 5 dorsal and 2 ventral teeth (*n* = 1), or 4 dorsal and 5 ventral teeth (*n* = 1) or 4 dorsal and 4 ventral teeth (*n* = 2). No studied specimen displayed a dorsal teeth or spine posterior to the postorbital margin. The number of carpal segments on pereopod 2 (P2) varied from 26 to 32. The number of meral segments on the second pereopod (P2) varied from 13 to 23. In 23 individuals, there was a difference of one or more segments on carpus and meral segments between right and left P2, respectively. The number of spiniform setae/teeth on the ventrolateral margin of the merus of pereopods 3–5 (P3-5) varied between 5–9, 4–9, and 3–7 on P3, P4 and P5, respectively. Most individuals exhibited differences of one seta between right and left P3-4 but rarely on P5. The number of spiniform setae on the ventral margin of carpus of P3-5 varied between 5–9, 4–9, and 3–7, on P3, P4 and P5, respectively. Similar to the meri, most individuals exhibit differences of one or more seta between right and left P3-5 (Fig. 2).

**Table 1:** Variability in morphological characters of systematic relevance in shrimps (*Lysmata boggessi* Rhyne & Lin, 2006) collected from aquarium stores (*N* = 43).

Characters	Position	Mean	SD	Range
Rostral spines	Dorsal	4.93	0.26	4–5
	Ventral	4.19	0.87	2–6
Number of carpal segments P2	Left	28.9	1.05	26–31
	Right	29.19	1.33	27–32
Number of meral segments P2	Left	17.9	1.89	13–23
	Right	18.67	1.96	15–22
Number of ischium segments P2	Left	3.3	0.51	3–6
	Right	3.43	0.67	3–6
Number of propodal spines P3	Left	10.55	0.77	8–12
	Right	10.62	0.73	9–12
Number of carpal spines P3	Left	4.71	0.97	2–7
	Right	4.93	0.95	2–7
Number of meral spines P3	Left	7.33	1.16	5–9
	Right	7.57	0.91	6–9
Number of propodal spines P4	Left	10.1	1.19	6–12
	Right	10.14	0.81	8–12
Number of carpal spines P4	Left	4.61	1.20	2–7
	Right	4.24	0.96	3–7
Number of meral spines P4	Left	6.49	0.90	5–9
	Right	6.43	0.97	4–8
Number of propodal spines P5	Left	9.56	1.22	8–12
	Right	9.5	1.16	8–12
Number of meral spines P5	Left	5.13	0.94	3–7
	Right	5	0.93	3–7

The combination of morphological traits above indicates that all specimens traded in the aquarium industry sampled during this study belong to *L. boggessi* and not *L*. *wurdemanni*, as commonly believed and as indicated by aquarium dealers. Still, comparison of our material (aquarium store specimens) with descriptions of *L. wurdemanni* and other related sympatric species (Rhyne & Lin, 2006) demonstrates considerable overlap on almost all characters analyzed (Table 1 and Table S1). For instance, specimens bought at aquarium stores resemble *L. boggessi* but also *L. wurdemanni, L. pederseni,* and *L. anchisteus,* with respect to the number of teeth in the dorsal rostrum (Table 1 and Table S1). Similarly, the overlap in the number of ventral rostral teeth between our aquarium store specimens, *L. boggessi*, *L. wurdemanni*, *L. pederseni*, *L. ankeri*, *L. bahia*, *L. rathbunae* and *L. udoi* is considerable. Major overlap in other characters between the aquarium store specimens and other closely related species, including the number of carpal segments on the second pereopod, and the number of spiniform setae on the ventral margin of the merus and propodus of pereopods 3, 4, and 5 are evident in Table S1. Importantly, limited information on character range, as well as the range of variation above, limits our ability to unequivocally identify the studied specimens as *L. boggessi* (see Table S1).

**Figure 3:**
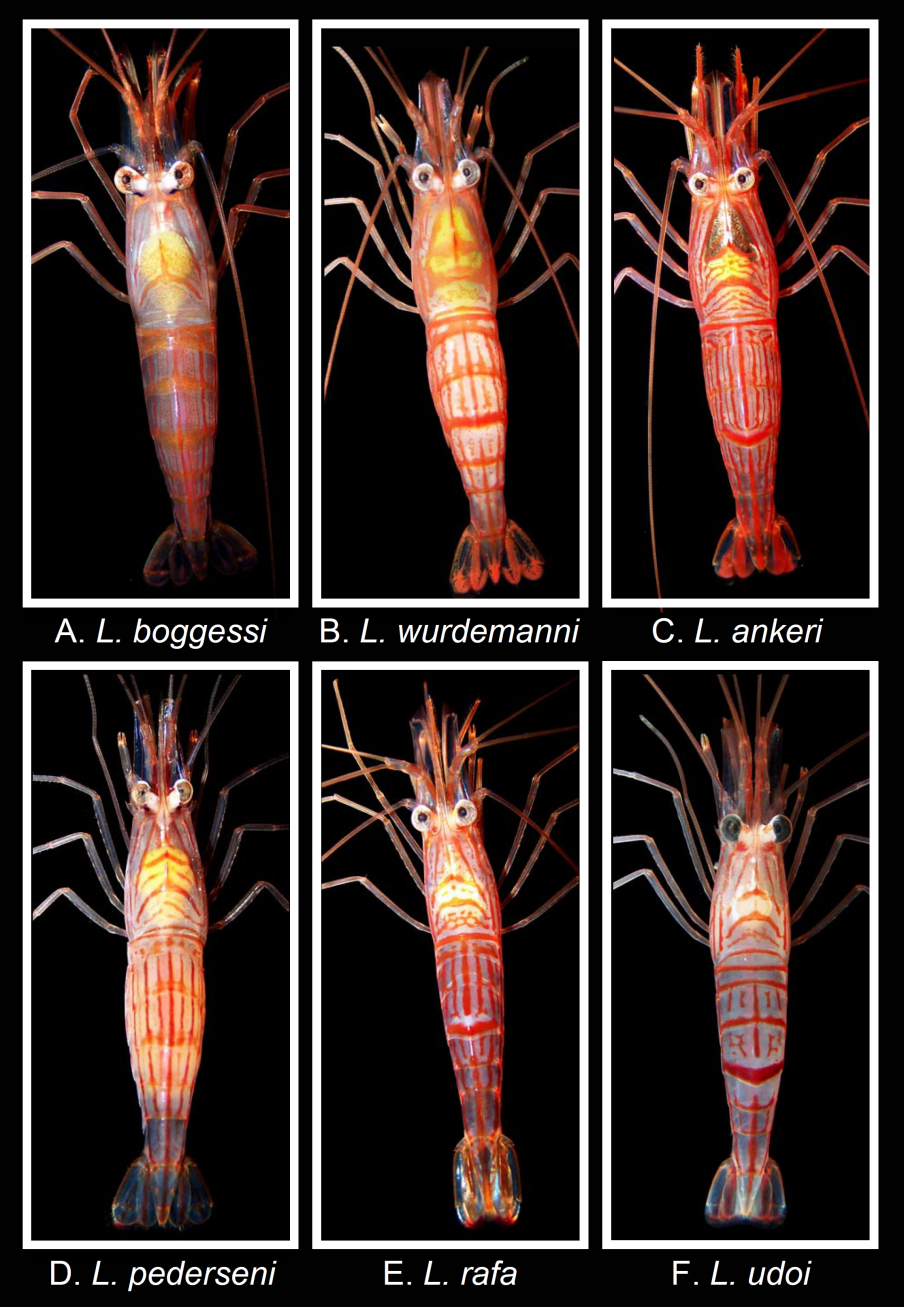
Coloration and color pattern (dorsal view) in shrimps belonging to the genus *Lysmata* from the Gulf of Mexico, western Atlantic, and Caribbean Sea. Photographs by J. Antonio Baeza. (A) *Lysmata boggessi*, (B) *Lysmata wurdemanni*, (C) *Lysmata ankeri*, (D) *Lysmata pederseni*, (E) *Lysmata rafa*, (F) *Lysmata udoi*.

### Coloration, color pattern and comparison with sympatric species

Contrary to the morphological characters, the coloration and color patterns of the aquarium store specimens fit with that reported for *L. boggessi* and is remarkably different from that reported for *L. wurdemanni* and other closely related species. Each specimen bought from an aquarium store exhibited a semi-translucent reddish exoskeleton covered with narrow, longitudinal, transverse, and oblique pale red stripes. The carapace exhibited V- and U-shaped oblique and transverse stripes. A distinctive inverted Y was observed on the carapace in dorsal view (Fig. 3). The abdominal pleura exhibited very narrow longitudinal stripes situated between broader and more intense longitudinal stripes. Importantly, the third pleuron lacked a red transverse band. Lastly, the telson and uropods exhibited a dark blue coloration (Fig. 3).

The coloration and color pattern above is highly dissimilar from that exhibited by *L. wurdemanni* and all other closely related species living in sympatry. In particular, *L. wurdemanni* bears a semi-translucent reddish exoskeleton with red longitudinal, transverse, and oblique bands distributed around the body, a carapace with broad transverse and oblique v-shaped bands; abdominal pleura with short, narrow longitudinal stripes, third pleuron with a broad transverse band (appearing more intense than bands of other pleura in dorsal view, and a telson and uropods with broad, intense longitudinal bands (Fig. 3, also, see Rhyne & Lin, 2006).

### Phylogenetic position of aquarium store specimens

**Figure 4:**
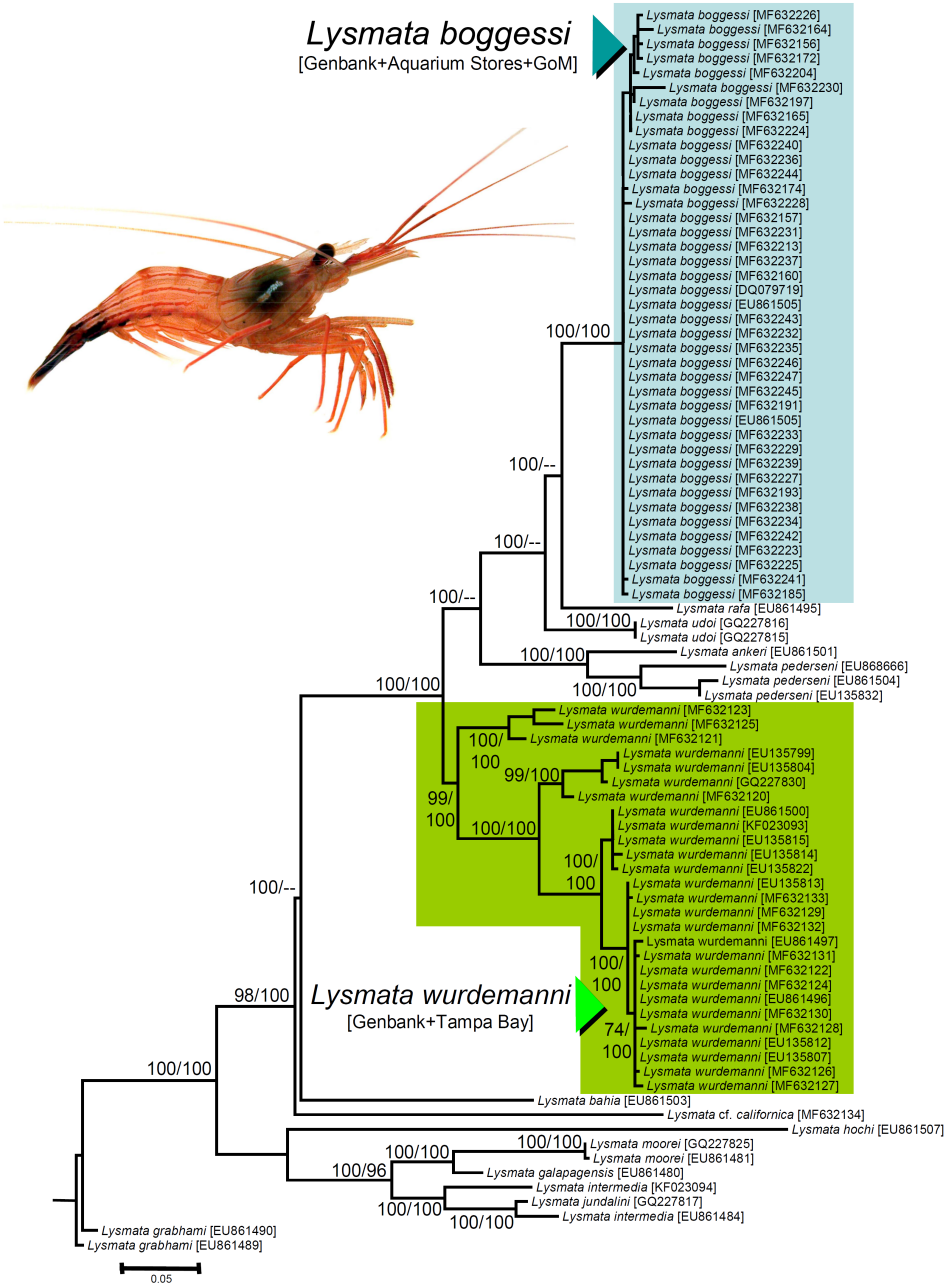
Phylogenetic tree obtained from Bayesian Information analysis (BI) of the partial 16S rRNA gene for the genus *Lysmata*, including the specimens bought at aquarium stores in southeastern USA, *Lysmata wurdemanni* (*N* = 27 specimens), *Lysmata boggessi* (*N* = 15 specimens), and other closely related species. Numbers above or below the branches represent the bootstrap values obtained from maximum likelihood (ML) and the posterior probabilities (multiplied by 100) from the BI analysis (ML/BI). Nodes with ML bootstrap values and BI posterior probabilities below 50 and 0.5(×100), respectively, are not shown.

We obtained nucleotide sequences of a 538–540 bp section of the 16S rRNA DNA gene from the 25 haphazardly chosen specimens bought from aquarium stores and reliably identified as *L. boggessi* based on coloration and color pattern (see above). All the sequences were a close match (Tamura Nei distance range = 0.002–0.018 not including identical sequences). In our phylogenetic analysis, the final aligned molecular data matrix was comprised of 651 characters, of which 213 were parsimony informative, for a total of 84 specimens from the Gulf of Mexico, Caribbean Sea, and northeastern Atlantic, belonging to peppermint shrimps from the genus *Lysmata* and two outgroup terminals belonging to the cleaner shrimp *Lysmata grabhami* (Fig. 4). Both molecular phylogenetic trees obtained with two different inference methods (ML and BI) resulted in the same general topology (Fig. 4). In the two phylogenetic analyses, the totality of the specimens bought from aquarium stores and all three sequences belonging to *L. boggessi* obtained from Genbank and from our population genetic study (see methods) clustered together into a single monophyletic clade strongly supported by a high posterior probability obtained from the BI analysis and was very well supported by the bootstrap support values from the ML analysis (Fig. 4). Also, all 27 sequences belonging to *L. wurdemanni* obtained from Genbank plus those retrieved from Tampa Bay clustered together into a second monophyletic clade strongly supported by the BI and ML analyses (Fig. 4).

Based on the geographic region that we sampled and the phylogenetic analyses above, the species most commonly traded in the aquarium ornamental industry is not *L. wurdemanni*, but instead is the congeneric *L. boggessi*, a species endemic to the eastern Gulf of Mexico.

### Population genetics of the endemic *Lysmata boggessi* in the Gulf of Mexico

Using 539 aligned sites, we found 13 different haplotypes among the 88 individuals of *L. boggessi* sampled across the eastern Gulf of Mexico. The number of haplotypes and haplotype diversity were similar across sampled localities (Table 2). The number of polymorphic sites and nucleotide diversity were similar among all the different studied localities but Cedar Key, which exhibited slightly greater values for the parameters above compared to the rest of the studied localities (Table 2).

**Table 2:** Standard diversity measures for populations of *Lysmata boggessi* in the eastern Gulf of Mexico. Shown for each population is the number of specimens collected at each site, the number of haplotypes (Nh), the number of polymorphic sites (Np), haplotype diversity (Hd), and nucleotide diversity (pi). Values in Hd and pi are presented as average ± standard deviations.

Locality	N	Nh	Np	Hd	pi
Sandy Key	18	3	3	0.451 ± 0.117	0.00157 ± 0.00043
Cedar Key	16	4	11	0.442 ± 0.145	0.00412 ± 0.00182
Hernando Beach	21	5	4	0.352 ± 0.131	0.00087 ± 0.00037
Pavilion Key	18	5	6	0.405 ± 0.143	0.00142 ± 0.00062
Sawyer Key	15	4	7	0.371 ± 0.153	0.00180 ± 0.00109

**Figure 5:**
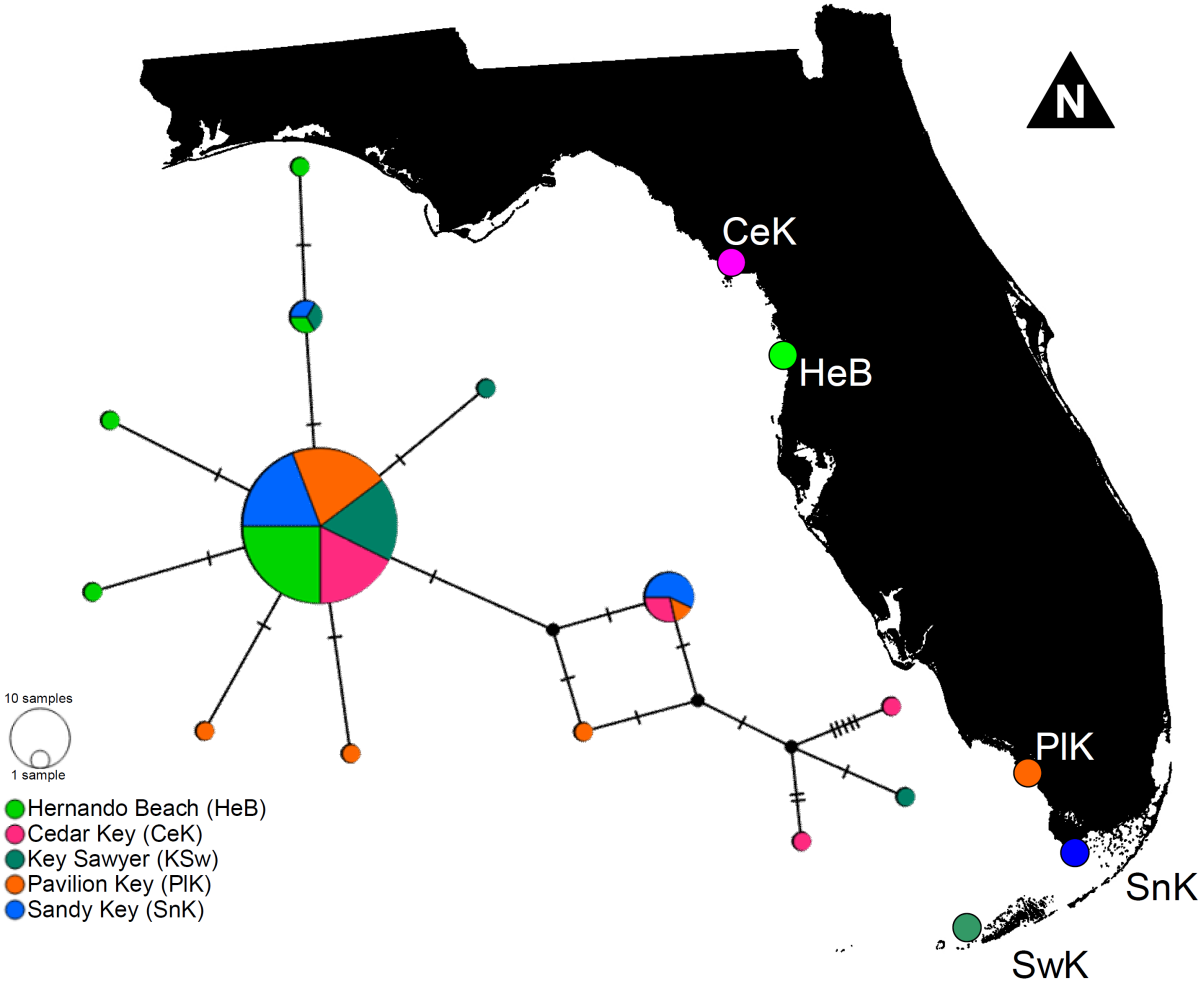
Minimum parsimony haplotype network for the 16S sequences of *Lysmata boggessi* in the eastern Gulf of Mexico. Each line separating two circles indicates a single substitution. The area of each circle corresponds to the number of haplotypes it represents. The color of the circle represents the location where the haplotype was found.

The haplotype network denoted only minor genetic structuring in *L. boggessi*, given that the 13 different haplotypes found among the 88 shrimps sampled during this study did not segregate together and formed distinguishable haplotype groups according to geographical location. A single high-frequency haplotype sequence was shared among all studied populations (Fig. 5). Two other sequences that were less common were shared among three of the five sampled populations. Hernando Beach and Pavillion Key contained the largest number of singleton haplotypes (*N* = 3). Cedar Key and Sawyer Key each contained two singleton haplotypes, while no singleton haplotypes were found at Sandy Key (Fig. 5).

In line with the haplotype network results, the AMOVA used to test for hierarchical population structure revealed a mean overall *F*_ST_ value of 0.02497. Molecular variation was much greater within than among populations (97.5% and 2.5%, respectively). As expected, the observed variability among populations was not significant and our data failed to reject the null hypotheses that *L. boggessi* populations are genetically homogeneous in the Gulf of Mexico (*P* = 0.0753).

A Mantel test did not reveal isolation by distance in *L. boggesssi* throughout the studied geographic range in the Gulf of Mexico (*r* = 0.3805, Mantel test: *P* = 0.9343). The reduced major axis regression (after log–log transformation of the variables) indicated that only 14.5% of total genetic variation was explained by geographic distance among Gulf of Mexico populations of *L. boggessi*.

## Discussion

### Market composition of the peppermint shrimp aquarium trade

Relying upon an integrative taxonomy approach, our public market survey found that *L. boggessi* was the only species sold at the aquarium trade market in the southeastern USA. This was a rather unexpected finding considering the public belief and information from aquarium dealers indicating that *L. wurdemanni* was the most traded species in the market (Calado, 2008; Rhyne & Lin, 2006; Rhyne et al., 2009). Studies measuring the extent of misidentification in other ornamental industries, either targeting marine, estuarine, or freshwater vertebrate and invertebrate organisms, are rare (e.g., Murray et al., 2012; Forsman et al., 2015; Johnston et al., 2017). If we compare our results to those from other industries experiencing the same problem, we found that the misidentification rate in the peppermint aquarium trade (100%) is among the highest estimated (i.e., misidentification reaches 97% in ‘Luo Gai Yu’ fish products purchased from Chinese (Nanjing and Shanghai) markets—Xiong et al., 2016b; 77% in ‘Chilean Sea Bass’ fillets from a certified sustainable fishery—Marko, Nance & Guynn, 2011; >66% in Cod purchased from Chinese (Nanjing and Shanghai) markets—Xiong et al., 2016b; 38% in Porgies species (family Sparidae) from an Italian (Milan) market—Armani et al., 2015; <10% in convenience (i.e., fish fingers) food in north-western England markets—Huxley-Jones et al., 2012. Several situations have been proposed to explain misidentification in other industries, from accidental product misidentification (Huxley-Jones et al., 2012) to voluntary misrepresentation in order to commit fraud in high-quality products for which the economic gains associated with mislabeling are large (Xiong et al., 2016a).

We believe that misidentification in the peppermint shrimp aquarium trade is accidental and involuntary, and is most likely explained by the variability in the diagnostic traits used to distinguish among peppermint shrimps and the remarkable visual similarity among species belonging to the genus *Lysmata* (Rhyne & Lin, 2006; Baeza, 2009). Our study represents a vivid example of how difficult it is to identify and distinguish among the different species of *Lysmata* inhabiting the western Atlantic, Gulf of Mexico, and Caribbean Sea (see Baeza, 2009). Indeed, our analysis based solely on anatomical diagnostic characters was not fully reliable in revealing *L. boggessi* as the most common species in the trade, but when the color pattern or genetic markers were also employed, it was possible to reliably identify the species present. The remarkable morphological similarity among the species belonging to the genus *Lysmata* makes species identification in this clade complicated, even to the expert (see Laubenheimer & Rhyne, 2010; Soledade et al., 2013). We argue in favor of additional studies using an integrative taxonomic approach similar to that used here to reveal public market composition in other ornamental industries.

Characterization of the composition of species in the market place in the peppermint shrimp aquarium trade has several implications. First, our study suggests that any effort towards producing basic life history information that can help to attain the goal of sustainability in this fishery needs to be directed towards *L. boggessi* and not *L. wurdemanni*. Considering how remarkably the ornamental industry has intensified during the last two decades, and the likelihood that it will continue to increase, it is imperative that we develop a baseline of biological information for many ornamental species so that fisheries for them can be managed sustainability (Wood, 2001; Green, 2003; Calado et al., 2003; Rhyne et al., 2009). Herein, we have conducted a literature review that reveals how little we know about the life history, ecology, behavior, and physiology of *L. boggessi*, especially when compared to *L. wurdemanni* (Table S2). For instance, individual- and population-level reproductive parameters are well documented in *L. wurdemanni* (Bauer & Holt, 1998; Bauer, 2002; Baldwin & Bauer, 2003; Baeza, 2006), but the same type of information is scant for *L. boggessi* (Baeza et al., 2014). The first studies on the life history of *L. boggessi* are underway. Similarly, information on population genetics was, until now, non existent for *L. boggessi* but population structure is relatively well known for *L. wurdemanni* (Rhyne et al., 2009). Considering that the inclusion of genetic research helps advancing management policies and conservation measures in fished resources, we studied the population genetics of the endemic *L. boggessi*.

### Population genetics of the endemic *Lysmata boggessi* in the Gulf of Mexico

Using sequences of the mitochondrial gene fragment 16S, we found no indication of population genetic structure in the endemic *L. boggessi* from the eastern Gulf of Mexico. Only a non-significant signal of genetic isolation by distance was found across the studied geographical range. The available information can explain the broadly shared haplotypes in this endemic species that exhibit a geographical range that extends for >550 km of lineal coast.

*Lysmata boggessi* exhibits a relatively long pelagic larval duration (approximately 24–36 d under laboratory conditions—A Rhyne, pers. comm., 2012), which suggests a high potential for population connectivity within a single generation. In the Gulf of Mexico, the Loop Current is the dominant ocean circulation feature that transports warm Caribbean water through the Yucatan Channel between Cuba and Mexico (Leben, 2005). The current most often bulges into the Gulf of Mexico, flows northwestward, and then loops anticyclonically to exit through the Straits of Florida, becoming the Florida Current (Leben, 2005). The Loop Current is one of the fastest currents in the Atlantic Ocean, exceeding 1 m/s (Dukhovskoy et al., 2015). Taking into account current speeds, the distance between Cedar Key and Sawyer Key (*L. boggessi* northernmost and southernmost populations, respectively), and the larval period in this species, larvae hatched at the northern edge of its geographic range need only 12–15 d to be passively transported to the southern edge of their geographic range. This period of putatively passive larval transportation is much shorter than the entire geographic range reported for this endemic species. This estimated transport time suggests that oceanographic conditions could facilitate larval transport throughout the entire geographic range of *L. boggessi* and explain its genetic homogeneity.

At first glance, the genetically uniform nature of *L. boggessi* in the Gulf of Mexico supports the long-standing view that mode of development in benthic marine invertebrates affects genetic structure: species with indirect larval development generally exhibit low spatial genetic structure relative to closely related species with direct or abbreviated development (e.g., Collin, 2001). Nonetheless, an increasing number of empirical studies and recent meta-analyses do not support the notion of a negative correlation between pelagic larval period and genetic structure in marine organisms (e.g., Bradbury et al., 2008; Weersing & Toonen, 2009; Riginos et al., 2011; Liggins et al., 2016). Indeed, the view that marine species with long pelagic larval durations have genetically uniform populations has been deemed a flawed generality for more than a decade (Selkoe & Toonen, 2011; Faurby & Barber, 2012). Contemporary and historical processes acting in parallel can produce unexpected genetic patterns (including short-scale spatial population structure in species with long larval periods—Hohenlohe, 2004; Soltis et al., 2006; Marko et al., 2010) and recent studies suggest that differences in the ecology are also important in explaining spatial genetic structure in species with long (and short) dispersal ability (Marko, 2004). Considering this, we interpret our results with caution because of the significant effects that marker type and number, sampling site density, sample size, and study design can play in determining whether or not isolation-by-distance and a relationship between larval duration and population genetic structure can be detected (Selkoe & Toonen, 2011; Faurby & Barber, 2012).

Certainly, a biophysical-based modeling approach (Cowen et al., 2000; Cowen, Paris & Srinivasan, 2006) can help determine connectivity patterns of *L. boggessi* populations in the eastern Gulf of Mexico with more detail and reveal if the *L. boggessi* stock in the eastern Gulf of Mexico is genetically-connected and homogeneous. Furthermore, we strongly argue in favor of additional studies using more powerful markers (SNPs generated by genome-wide genotyping—Wang et al., 2012; Toonen et al., 2013) capable of revealing genetic structure at a finer spatial-scale given the observed isolation by distance signal we detected in this species. It would also be valuable in revealing source–sink meta-population dynamics in *L. boggessi*, for which our data was not sufficient. For example, determining if the northernmost and southernmost populations represent sources and sinks, respectively, for *L. boggessi* remains to be addressed by integrative studies measuring population-level reproductive performance, modeling approaches to estimate connectivity across the geographic range of this shrimp, and genetic markers capable of resolving finer scale genetic structure.

## Conclusions

Relying upon an integrative taxonomy approach, a public market survey revealed that *L. boggessi*, an endemic species to the eastern Gulf of Mexico, was the only species sold at the aquarium trade market in the southeastern USA during our study period. Taking into account that the southeastern USA is the most intense market for peppermint shrimps in the world, *L. boggessi* is probably also the most traded peppermint shrimp worldwide. The use of color patterns and molecular characters were crucial to reliably and unequivocally identify the species in the trade and to distinguishing *L. boggessi* from other closely related congeners. We argue in favor of additional studies using a similar integrative approach to measure the extent of misidentification at the market place in other ornamental fisheries. Misidentification in the peppermint shrimp trade is likely accidental and involuntary, probably arising from the variability in diagnostic traits used to distinguish among species and the remarkable visual similarity among species of the genus *Lysmata*. The population genetic analysis of *L. boggessi* failed to reject the null hypotheses that populations are genetically homogeneous, suggesting there is a single stock across the east Gulf of Mexico. However, given the observed isolation by distance signal we detected, additional studies should be conducted using more powerful markers (e.g., SNPs) capable of revealing genetic structure at a finer spatial scale. Overall, the information generated here will improve identification of the species in the marketplace, help guide comprehensive management measures, and underscores the need to have basic population information on a species to achieve the goal of sustainable harvest.

##  Supplemental Information

10.7717/peerj.3786/supp-1 Table S1 Morphological characters of systematic relevance and color pattern of shrimps of the genus *Lysmata* collected from aquarium stores and the closest congeneric species

10.7717/peerj.3786/supp-2 Table S2 Information on the life history of *Lysmata boggessi* and *L. wurdemanni*

10.7717/peerj.3786/supp-3 Data S1 Raw data Morphological variability in Lysmata boggessi from the Aquarium Trade.

## Additional Information and Declarations

### Field Study Permissions

The following information was supplied relating to field study approvals (i.e., approving body and any reference numbers): Field collections were approved by FWCC (permit number: SAL-11-1319-SR).

### DNA Deposition

The following information was supplied regarding the deposition of DNA sequences: The 16S sequences described here are accessible via GenBank accession numbers MF632120–MF632134 and MF632135–MF632247.

### Data Availability

The following information was supplied regarding data availability: The raw data has been provided as a Supplemental File.
