# Supplementary material for: Integrative taxonomy of the ornamental ‘peppermint’ shrimp public market and population genetics of Lysmata boggessi, the most heavily traded species worldwide
Source: PeerJ. 2017 Sep 18;5:e3786. doi: 10.7717/peerj.3786 (PMC5607919; doi:10.7717/peerj.3786)
Supplement: Table S1 [file peerj-05-3786-s001.doc]

**Supplementary Table .** Morphological characters of systematic relevance and color pattern of shrimps of the genus *Lysmata* collected from aquarium stores and the closest congeneric species.

| **Characters** | ***Lysmata* (trade)** | ***L. boggessi*** | ***L. wurdemanni*** | ***L. pederseni*** | ***L. ankeri*** | ***L. bahia*** | ***L. rathbunae*** | ***L. anchisteus*** | ***L. rafa*** | ***L. udoi*** |
| --- | --- | --- | --- | --- | --- | --- | --- | --- | --- | --- |
| N dorsal rostral teeth | 4 – 5 | 3 – 6 | 4 – 6 | 7 – 8 | 6 – 8 | 6 – 7 | 5 – 6 | 5 – 6 | 7 | 6 |
| N dorsal rostral teeth posterior to the orbit | 2 | 2 | 2 | 2 | 2 | 2 | 2 | 1 | 2 | 2 |
| N ventral rostral teeth | 2 – 6 | 3 – 5 | 2 – 6 | 5 – 7 | 4 – 6 | 3 – 4 | 3 – 5 | 1 | 7 – 9 | 5 |
| Rostrum length | 0.6 – 0.8 times as long as carapace | 0.6 – 1.0 times as long as carapace | 0.4 – 0.7 times as long as carapace | 0.7 – 1.1 times as long as carapace | 0.6 – 0.8 times as long as carapace | 0.5 times as long as carapace | Reaching beyond the antennular peduncle | Reaching beyond the antennular peduncle | 1.2 times as long as carapace | 0.9 times as long as carapace |
| Pterygostomial tooth | Absent | - | - | - | - | - | - | - | Absent | Absent |
| Accessory branch of dorsal antennular flagellum | Rudimentary with second ramus |  | Rudimentary with secondary ramus |  |  |  |  |  | Secondary ramus reduced to rudiment | Accessory branch of outer ramus rudimentary |
| N carpal segments on pereopod 2 | 26 – 32 | 25 – 32 | 27 – 32 | 33 – 41 | 33 – 41 | 29 – 31 | 30 – 35 | 13 – 15 | 40 – 43 | 33 |
| N meral segments on pereopod 2 | 13 – 23 |  | 15 |  |  |  | 16 – 24 | 7 | 25 | 20 |
| N ischium segments on pereopod 2 | 3 – 6 |  |  |  |  |  | 3 – 5 | 1 – 2 |  |  |
| N spiniform setae on ventral margin of merus / propodus of pereopod 3 | 5 – 9 /  8 – 12 |  |  |  |  |  | 5 – 8 /  6 | 1 / 6 |  | 7 / - |
| N spiniform setae on ventral margin of merus / propodus of pereopod 4 | 4 – 9 /  6 – 12 |  |  |  |  |  | 4 – 7 /  - |  |  |  |
| N spiniform setae on ventral margin of merus / propodus of pereopod 5 | 3 – 7 /  8 – 12 | 3 – 6 /  - | 1 – 4 / | 3 – 6 /  - | 0 – 6 /  - | 1 – 6 /  - | 3 – 5 /  - | - /  5 |  |  |
