# Supplementary material for: Integrative taxonomy of the ornamental ‘peppermint’ shrimp public market and population genetics of Lysmata boggessi, the most heavily traded species worldwide
Source: PeerJ. 2017 Sep 18;5:e3786. doi: 10.7717/peerj.3786 (PMC5607919; doi:10.7717/peerj.3786)
Supplement: Table S2 [file peerj-05-3786-s002.doc]

Supplementary Table 2. Information on the life history of *Lysmata boggessi* and *L. wurdemanni*.

| Biological Parameter |  | *L. boggessi* | *L. wurdemanni* | Authority |
| --- | --- | --- | --- | --- |
| Embryo development |  | ? | ? |  |
| Larval development |  | Completed | ? | A. Rhyne. pers. com. |
| Size at (male) first maturity |  | ? | ? |  |
| Size at sex change (CL, mm) |  | 6.1 - 7.4 | 6.6 - 10.4 | Baldwin and Bauer 2003, Baeza et al. 2014 |
| Age at sex change (years) |  | ? | 0.27 - 1.07 | Baldwin and Bauer 2003 |
| Sex Phase Ratio  (males / total individuals) |  | 0.46 - 0.79 | 0.14 - 0.88 | Baeza 2007, Baeza et al. 2014 |
| Growth Rate (mm / year) |  | ? | 4.0 - 6.7 | Baldwin and Bauer 2003 |
| Mating behavior |  | ? | Observed | Bauer and Holt 1998 |
| Fecundity  (eggs / female) |  | 554 - 829 | 500 - 1500 | Baeza 2007, Baeza et al. 2014 |
| Reproductive output  (egg mass / body mass, %) |  | 13.6 - 17.7 | ? | Baeza et al. 2014 |
| Egg Size  (volume, mm^3^) |  | 0.07 - 0.08 | ? | Baeza et al. 2014 |
| Lifespan (years) |  | ? | 1.02 - 1.06 | Baldwin and Bauer 2003 |
| Population dynamics |  | ? | Reported | Bauer 2002 |
| Reproductive Season (month rage) |  | ? | Feb - Oct | Baldwin and Bauer 2003 |
| Social Structure |  | ? | Gregarious | Bauer and Holt 1998 |
| Habitat |  | Seagrass Beds | Rocky intertidal | Baeza et al. 2014 |
| Predators |  | ? | *Scartella cristata* | Baeza 2006 |
| Parasites |  | *Parabopyrella lata* | *Parabopyrella* sp. | Boyko 2006 |
|  |  |  |  |  |
